# Impact of anesthesia and analgesia techniques on glioblastoma progression. A narrative review

**DOI:** 10.1093/noajnl/vdaa123

**Published:** 2020-09-16

**Authors:** Ann Privorotskiy, Shreyas P Bhavsar, Frederick F Lang, Jian Hu, Juan P Cata

**Affiliations:** 1 Eastern Virginia Medical School, Norfolk, Virginia, USA; 2 Department of Anesthesiology and Perioperative Medicine, The University of Texas MD Anderson Cancer Center, Houston, Texas, USA; 3 Department of Neurosurgery, The University of Texas MD Anderson Cancer Center, Houston, Texas, USA; 4 Department of Cancer Biology, The University of Texas MD Anderson Cancer Center, Houston, Texas, USA

**Keywords:** anesthetic, analgesic, glioblastoma, progression

## Abstract

Glioblastoma (GBM) is an aggressive malignant CNS tumor with a median survival of 15 months after diagnosis. Standard therapy for GBM includes surgical resection, radiation, and temozolomide. Recently, anesthetics and analgesics have received attention for their potential involvement in mediating tumor growth. This narrative review investigated whether various members of the 2 aforementioned classes of drugs have a definitive impact on GBM progression by summarizing pertinent in vitro, in vivo, and clinical studies. Recent publications regarding general anesthetics have been inconsistent, showing that they can be pro-tumoral or antitumoral depending on the experimental context. The local anesthetic lidocaine has shown consistent antitumoral effects in vitro. Clinical studies looking at anesthetics have not concluded that their use improves patient outcomes. In vitro and in vivo studies looking at opioid involvement in GBM have demonstrated inconsistent findings regarding whether these drugs are pro-tumoral or antitumoral. Nonsteroidal anti-inflammatory drugs, and specifically COX-2 inhibitors, have shown inconsistent findings across multiple studies looking at whether they are beneficial in halting GBM progression. Until multiple repeatable studies show that anesthetics and analgesics can suppress GBM growth, there is no strong evidence to recommend changes in the anesthetic care of these patients.

**Key Points**

- General anesthetics and opioids do not impact survival after glioblastoma surgery.
- Regional anesthesia is not associated with improvement in survival after glioblastoma surgery.
- More studies are needed to elucidate if perioperative use of COX-2 inhibitors impact survival after glioblastoma surgery.

Gliomas are the most common type of malignancy in the CNS. These cancers arise from tumors in neural support cells such as astrocytes and oligodendrocytes. Glioblastoma (glioblastoma multiforme/GBM) is a type of very aggressive glioma and is associated with a poor prognosis.^1^ Current guidelines recommend gross total resection of the enhancing solid tumor followed by radiation and the oral alkylating chemotherapy agent temozolomide (TMZ).^1,2^ However, therapeutic strategies should be tailored based on age and functional status.^3^ Disease progression is the most common cause of death in patients with GBM; therefore, understanding the mechanisms and risk factors involved in tumor growth postoperatively has been the focus of extensive laboratory and clinical investigations.^4^

In recent years, there has been a growing interest in determining the impact of anesthetics and analgesics in cancer progression. One theory is that short-term exposure to anesthetics, such as volatile anesthetics, could be associated with accelerated tumor growth.^5^ Similarly, it has been proposed that patients who are exposed to opioids in large quantities for days after surgery would suffer cancer progression more rapidly than those taking limited amounts of analgesics and for a shorter period.^6^ Lastly, researchers have speculated that regional anesthesia could have a beneficial impact on oncological outcomes since it could minimize opioid use, reduce the consumption of general anesthetics, and limit the stress response associated with surgery (Figure 1).^7^

**Figure 1.**
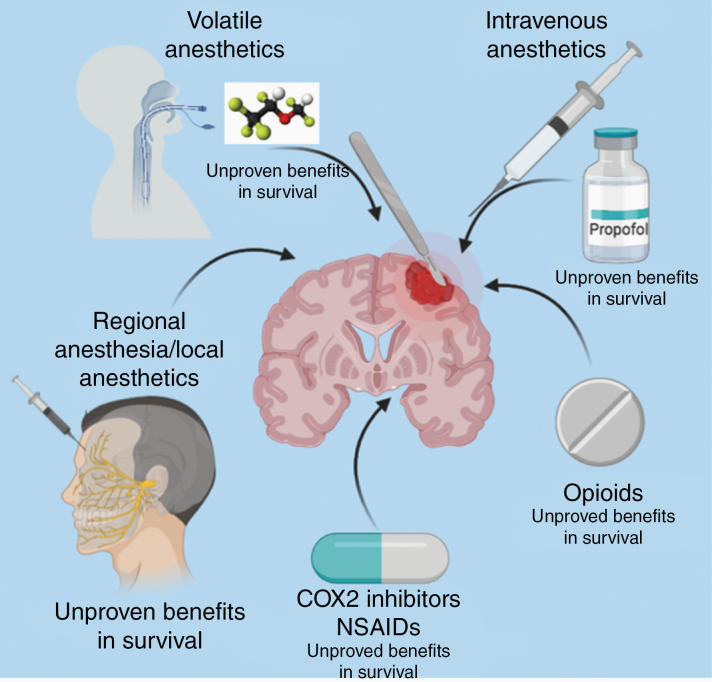
The pharmacological interventions speculated to affect prognosis in patients with glioblastoma (GBM). Clinical studies indicate that general anesthetics and opioids do not have a significant impact on GBM progression. The role of COX-2 inhibitors and NSAIDs on GBM progression has not been well studied in the context of surgery.

In this narrative review, we summarized the current laboratory and clinical evidence on the impact of different anesthetic and analgesic techniques on tumor progression.

## Glioblastoma: An Overview

GBM is the most commonly occurring primary adult malignant CNS tumor in the United States, accounting for 48.3% of tumors in this category. With an incidence rate of 3.22 per 100,000 persons in the United States, this tumor is more common in males than females (1.6:1). Incidence rates are approximately twice as high in Whites than in Blacks.^8^ GBM is primarily diagnosed at a median age of 65 years old, and the incidence increases with age. GBM’s 5-year relative survival rate is the lowest (6.8%) compared with the 5-year relative survival rate of all other malignant brain tumors.^8^ Thus, the prognosis is poor, with a median survival of 15 months after diagnosis is made.^4^

In 2016, the World Health Organization (WHO) reclassified CNS tumors based not only on their histologic features but also on their molecular parameters. This restructuring changed how clinicians diagnose and treat CNS tumors. GBM remained classified as a grade IV tumor, corresponding to the highest degree of malignancy, and was subclassified into the following 3 categories: isocitrate dehydrogenase (IDH)-wildtype, IDH-mutant, and not otherwise specified (NOS).^4^ IDH-wildtype is the most common variant of this disease, accounting for 90% of GBM cases. This type of GBM corresponds with primary/de novo GBM, occurs predominantly in patients over the age of 55 years, and has a wide anatomical distribution potential in the brain. IDH-mutant GBM accounts for the remaining 10% of all GBM cases and corresponds with secondary GBM. This type of malignant glioma is thought to have arisen from a low-grade astrocytoma, in contrast with primary GBM. IDH-mutant GBMs occur predominantly in a younger population (<55 years old), have a better prognosis than IDH-wildtype GBM, and are preferentially located in the frontal lobe. The diagnosis of NOS GBM should be reserved for scenarios in which IDH evaluation cannot be performed.^9,10^ The cIMPACT-NOW (the Consortium to Inform Molecular and Practical Approaches to CNS Tumor Taxonomy) was established to rapidly integrate advances in brain tumor molecular pathogenesis into clinical practice. A recent recommendation from the CIMPACT-NOW is to designate diffuse, grade II or III, IDH-wildtype astrocytomas with molecular features of GBM directly as “Glioblastoma, IDH-wildtype.” ^11^

### Genetic and Molecular Alterations

Three signaling pathways are commonly deregulated in GBMs: loss of function of RB, loss of function of p53, and gain of function of RTK/RAS/PI3K.^12^ Furthermore, certain genetic alterations have been found helpful in differentiating primary and secondary GBM tumors. Mutations commonly found in primary GBM include epidermal growth factor receptor (*EGFR*) overexpression and amplification, *MDM2* amplification, deletion of p16, loss of heterozygosity (LOH) of chromosome 10q, and *PTEN* mutations. Mutations commonly found in secondary GBM include LOH of 19q, mutations in RB, *CDK4* amplification, and overexpression of platelet-derived growth factor (*PDGF*) *PDGFA/PDGFRa*.^13^

GBMs can also be classified based on transcriptome profiles into the following 4 subgroups: classical, mesenchymal, proneural, and neural. The classical subgroup exhibits loss of *PTEN* and amplification of *CDKN2A* and *EGFR*. The mesenchymal subgroup typically has mutations and/or loss of *TP53*, *NF1*, and *CDKN2A*. This variant is associated with a less favorable prognosis and is more frequent in older patients. The proneural transcriptome subgroup is characterized by mutations in *CDK4*, *CDK6*, *PDGFRA*, *MET*, and *IDH1*. In contrast to the mesenchymal subgroup, GBMs identified to be proneural are associated with younger patients and have a more favorable prognostic outcome. Additionally, proneural GBMs are associated with secondary GBMs. The neural subtype does not have a hallmark genetic profile but commonly has expression of neuron markers such as *NEFL* and *GABRA1*.^14^ Alterations in the above pathways allow GBMs to evade cell-cycle checkpoints and proliferate uncontrollably. Additionally, some mutations confer resistance to apoptotic stimuli allowing for enhanced tumor cell survival. Increased understanding of genomic contributions to GBM pathogenesis can aid in identifying novel therapeutic targets.^15^

The microenvironment surrounding GBM cells plays a crucial role in tumor development, progression, prognosis, and response to adjuvant therapies. In the tumor niche, GBM cells release attractant factors to recruit endothelial cells (angiogenesis) and immunocytes, including glioma-associated microglia/macrophages and tumor-infiltrating lymphocytes (ie, CD4+ T cells, CD8+ T cells, natural killer [NK] cells, and regulatory T cells [Tregs]). Detailed analysis of the immunocyte content in nonrecurrent GBMs indicates an immunosuppressive microenvironment that is characterized by a predominance of glioma-associated macrophages (GAMs), T cells exhaustion, regulatory B cells, noncytolytic NK cells, and an increased number of Tregs.^16,17^ GAMs are critical cells in the tumor microenvironment because they participate in gliomagenesis, express checkpoints (ie, PD-1 and CD73) and can release immunosuppressive cytokines (ie, interleukin [IL]-6, IL-10 and transforming growth factor B).^18^ When Gordon et al. downregulated PD-1 in vivo, they observed enhanced phagocytic function in GAMs toward GBM cells and tumor reduction.^19^ Furthermore, GAMs can drive invasive T cells to functional exhaustion or anergy. As a result, novel therapies including checkpoint inhibitors have been developed to modulate GAMs, rescuing T cells from exhaustion and depleting Tregs.^20^

## Impact of Anesthetics and Analgesics on GBM Progression

### General Anesthetics

Surgical resection of GBM tumors can be performed under volatile-based general anesthesia or total intravenous anesthesia (TIVA). Volatile anesthetics such as isoflurane, sevoflurane, and desflurare are administered as the base of inhalational anesthesia, while propofol is the most commonly used anesthetic for TIVA. Other intravenous agents such as dexmedetomidine are also frequently added as adjuvant anesthetics during craniotomies for tumors. This is done to provide analgesia and reduce anesthetic consumption.^21^ The term “combined general anesthesia” refers to the combination of volatile anesthesia and propofol anesthesia during surgery.^22^ The goal of using a combined general anesthesia technique is to reduce the adverse effects of either single agent (ie, volatile anesthesia–induced nausea and vomiting).

Experimental studies investigated whether general volatile or intravenous anesthetics can either potentiate or inhibit tumor malignancy factors such as cell migration, invasion, and proliferation (Table 1). One proposed mechanism by which volatile anesthetics impact cancer outcomes is by mediating gene expression in tumor cells.^23^ For instance, short-term exposure of glioma cells to isoflurane and desflurane modulated gene expression in a time-dependent manner.^23^ Babateen et al. reported that the human GBM cell line U3047MG abundantly expressed functional GABA_A_ subunit receptors, and these receptors can be modulated by classic GABA-targeting anesthetics.^24^ Thus, different groups of researchers have investigated the impact of GABA-targeting anesthetics on GBM cell behaviors. Lai et al. found that 4% sevoflurane promotes pro-tumoral characteristics in human GBM cell lines U251, A172, and U87.^25^ Interestingly, a different study showed contrary findings; U251 and U87 cells exhibited decreased proliferation, invasion, and migration abilities after treatment with the same concentration of sevoflurane as previously described. The proposed mechanism behind these findings was via upregulation of microRNA(miR)-124-3p.^26^ The inconsistencies in these studies can be explained by different experimental conditions. The involvement of miRNAs in anesthetic antitumor effects was additionally observed in GBM cells treated with propofol. Remarkably, when glioma cells were treated with propofol, they showed inhibited cell proliferation, migration, and invasion, thought to be mediated at least in part via upregulation of miR-410-3p.^27^

**Table 1.** Summary of Preclinical and Clinical Studies Investigating the Effects of General Anesthetics on GBM Biology and Patients’ Survival

Author/Year	Study Design	Pertinent Findings
Lai (2019)	In vitro and in vivo (mice) Human GBM U251, U87 and A172 cells Cultured for 4 h 1%–4% sevoflurane	Sevoflurane exhibits pro-tumoral properties in GBM cells at least in part by upregulation of CD44.
Gao (2019)	In vitro Human GBM U251 and U87 cells. Cultured for 4 h with 4% sevoflurane	Sevoflurane exhibits antitumoral properties in glioma cells at least in part by modulation of the miRNA-124-3p/ROCK1 pathway.
Li (2020)	In vitro U251 and A172 cells 5 µg/mL or 10 µg/mL of propofol for 24 h	Propofol exhibits antitumoral properties in glioma cells at least in part by modulation of the miR-410-3p/TGFBR2 pathway.
Cata (2017)	Clinical retrospective study (*n* = 261 desflurane, n=117 isoflurane)	In the context of GBM surgery, neither isoflurane nor desflurane impact PFS and/or OS in patients. Median PFS and OS times were 8.84 (7.92–10.28) and 19 (17.31–22.93) months, respectively. At 5 years, the progression and overall mortality rate were 93% and 85%. Exposure to isoflurane exposure was longer than desflurane (*P* < .002).
Grau (2020)	Clinical retrospective study (*n* = 158)	The choice of inhaled anesthetics vs TIVA in surgical resection of GBM does not impact patient survival. No significant difference in recurrence-free survival (volatiles: 8.0 vs TIVA: 8.4 months; *P* = .54) or OS (propofol: 17.4 vs volatiles: 16.9 months; *P* = .85),
Dong (2019)	Clinical retrospective study (*n* = 154 propofol, *n* = 140 sevoflurane)	The choice of propofol vs sevoflurane in surgical resection of GBM does not impact patient survival. Median PFS was 10 months propofol vs 11 months sevoflurane. Median OS was 18 months propofol vs 18 months sevoflurane.

GBM, glioblastoma; miRNA-124-3p, microRNA-124-3p; miR-410-3p, microRNA-410-3p; OS, overall survival; PFS, progression-free survival; ROCK1, Rho-associated coiled-coil containing protein kinase 1; TGFBR2, transforming growth factor-β receptor type 2; TIVA, total intravenous anesthesia.

As previously mentioned, gain of function of the RTK/RAS/PI3K pathway is critical in GBM tumorigenesis.^12^ Volatile anesthetics are known modulators of RAS/PI3K signaling in different cancer cell lines including hepatocarcinoma, in which they have shown to promote apoptosis and inhibit migration.^28^ Volatile anesthetics can also regulate cell division by acting on p53 signaling. This was suggested by Ni et al. who reported that isoflurane inhibited the repair of DNA damage in human neuroglioma cells via the p53 signaling pathway.^29^ However, it is unknown whether the effects of volatile anesthetics on the RAS/PI3K and p53 pathways affect the behavior of GBM cells.

Volatile anesthetics and propofol appear to have opposite effects on the innate immune system. While volatile anesthetics inhibit the function of NK cells, propofol has some stimulatory effects on these cells.^30^ A decreased expression of the adhesion molecule leukocyte-associated antigen-1 has been proposed as one of the mechanisms by which isoflurane and sevoflurane suppress the function of NK cells.^31^ In contrast, propofol can stimulate the function of NK cells by increasing the expression of granzime B and IFNγ activating surface receptors (CD16, NKp30, NKp44, and NKG2D), and inhibiting prostaglandin E2 formation.^32–34^ It is worth considering that the immunomodulatory effects of general anesthetics have been evaluated in vitro in NK cells; therefore, it remains to be determined whether the function of NK cells and lymphocytes is altered in the GBM microenvironment in vivo.

When considering the impact of anesthetics on GBM progression, it is important to understand that the duration of exposure to these drugs is short (2–6 h) and dependent on the duration of surgery.^22^ As expected, longer operations are required for larger and/or more complex tumors; thus, impact of any anesthetic on tumor progression is significantly confounded by time of surgery. Also, the dosage of the anesthetic used during surgery is confounded by duration of surgery. The cumulative dose of agents like propofol is higher after longer surgeries. Our group reported that neither isoflurane nor desflurane had any impact on the progression-free survival (PFS) and overall survival (OS) rates in patients who had exposure to either of these anesthetics during craniotomy for GBM.^22^ More recently, 2 observational studies investigated the association between the use of TIVA versus volatile-based general anesthesia during GBM resection. The studies included 158 and 294 patients and concluded that the TIVA was not associated with any improvement in survival.^35,36^ These studies suggest that the method of delivering general anesthetics does not influence GBM progression after surgery.

### Local Anesthetics

Regional anesthesia is currently recommended for adequate perioperative pain control after craniotomy.^37^ It has been speculated that regional anesthesia could reduce cancer progression by various mechanisms acting synergistically, including via a reduction in opioid and general anesthetic use, modulation of the stress response, and the direct effect of local anesthetics on the tumor microenvironment after their systemic absorption.^38^ Based on these premises, the effects of local anesthetics in glioma cells have been investigated in vitro (Table 2).^39,40^ Overall, lidocaine has shown to induce apoptosis and autophagy in rat C6 glioma cells.^40^ Several mechanisms were proposed, including a blockade of current in transient receptor potential melastatin 7 channels with concentrations of lidocaine ranging from 1 to 3 mM and induction of alteration in the cellular cytoskeleton.^39,40^ In U87-MG glioma cells, lidocaine triggered dose-dependent apoptosis by increasing intracellular calcium concentrations.^41^ It is unknown how the in vitro findings translate in vivo. To the best of our knowledge, the effects of lidocaine or any other local anesthetics have not been tested in GBM animal models.

**Table 2.** Summary of Preclinical and Clinical Studies Investigating the Effects of Lidocaine and Regional Anesthesia on GBM Biology and Patients’ Survival

Author/Year	Study Design	Pertinent Findings
Izdebska (2018)	In vitro C6 rat glioma cells Treated for 24 h with 0.25, 0.5, 1, 5, 10, 15, and 30 mM of lidocaine	Lidocaine exhibits antitumoral effects in rat glioma cells at least in part by cytoprotective autophagy.
Leng (2017)	In vitro C6 rat glioma cells Treated with 1 and 3 mM lidocaine	Lidocaine exhibits antitumoral effects at least in part by inhibition of transient receptor potential melastatin 7 channels.
Lu (2016)	In vitro U87 cells Treated with 1, 5, 10, 20, and 40 mmol/L of lidocaine.	Lidocaine exhibits antitumoral effects at least in part by induction of apoptosis secondary to increased intracellular calcium ion concentration, and mitochondrial membrane potential.
Zheng (2017)	Clinical retrospective study (*n* = 119)	Use of scalp block in GBM patients undergoing tumor resection is associated with reduced inflammation and improved survival. Median PFS was 16.7 months in patients who had scalp block vs 6.5 months in those who did not (*P* < .001).
Cata (2018)	Clinical retrospective study (*n* = 808)	Use of scalp block in brain tumor resection is not associated with improved patient survival. Median PFS time was 7.69 months; 5-year PFS rate was 8%. Median OS time was 16.82 months; 5-year OS rate was 17%.

GBM, glioblastoma; OS, overall survival; PFS, progression-free survival.

Local anesthetics have shown antiangiogenic and immunomodulatory effects. Lidocaine administered systemically (30 mg/kg) to mice with melanoma decreased tumor growth by inducing apoptosis in endothelial cells.^42^ Furthermore, lidocaine in clinically relevant concentrations enhanced the function of NK cells and inhibited the production of IL-6, tumor necrosis factor (TNFα), and IL-12 from activated dendritic cells.^43,44^ In humans, the use of scalp blocks for GBM surgery was associated with a lower neutrophil to lymphocyte ratio, suggesting a modulatory effect of regional anesthesia on inflammation.^45^

While the effects of local anesthetics have not been evaluated in clinical trials, our group investigated whether the use of regional anesthesia has any effects on PFS and OS after surgery. Briefly, we were unable to demonstrate a beneficial association between the use of scalp blocks and progression-free or OS in a cohort of 534 patients.^46^ It remains unknown whether the intravenous infusion of lidocaine would have any impact on survival after GBM surgery.

### Opioids

Modulation via μ-opioid receptors has been theorized as adjuvant therapy in overcoming the enhanced chemo- and radioresistance in GBMs. Methadone has received particular attention regarding its potential role in GBM progression. Friesen et al. demonstrated that methadone reduced tumor cell malignancy by enhancing apoptosis and increasing sensitivity to treatment with doxorubicin in 2 GBM cell lines. Additionally, the study reported tumor growth inhibition in vivo in mice treated with methadone.^47^ Unfortunately, subsequent investigations showed that the antitumor effects of methadone are only exhibited at high concentrations that would not be clinically feasible in patients. ^48,49^ One study demonstrated that at elevated concentrations of this opioid, GBM cells exhibit increased apoptosis and reduced cell viability. However, when cells were treated with clinically relevant concentrations, methadone did not promote any antitumorigenic effects, and one cell line even showed higher proliferation after treatment compared to controls.^48^ Similarly, Opperman et al. reported reduced GBM cell viability only when treated with concentrations of methadone that have previously been reported to be toxic in patients. Additionally, the study showed that methadone did not improve tumor cell response when combined with the standard therapies of TMZ and/or radiation.^49^

In addition to methadone, enkephalin has also been investigated for potential roles in mitigating GBM. Biphalin, an enkephalin analog, has been of interest in recent years due to its less addictive nature and high analgesic potency as compared to morphine. Biphalin treatment resulted in antitumorigenic effects including reduced proliferation rates and colony formation in human GBM cells. Interestingly, morphine displayed the opposite effect and stimulated tumor cell proliferation.^50^ In a study by Heiss et al., activation of the μ-opioid receptor by enkephalin and etorphine (a synthetic analog of morphine) promoted tumor cell survival by inhibition of apoptosis in neuroblastoma × glioma hybrid cells.^51^ These effects were believed to be mediated by activation of the Akt pathway.^51^ Though a different cancer cell line was used in the study investigating the effects of biphalin, the tumor cytoprotective effects seen in the neuroblastoma × glioma hybrid cells after treatment with enkephalin and etorphine raise concern regarding the safety profile of similar opioids in the context of GBM.

The results of experimental studies demonstrate that opioids can impair mechanisms of immune surveillance and modulate inflammation. Forget et al. reported that the systemic administration of fentanyl (40 µg/kg) to rats undergoing surgery promoted metastasis.^52^ Similarly, when patients were treated with a large dose of fentanyl (75 µg/kg) during surgery, they had a prolonged (48 h) suppression of NK cell cytotoxicity compared to those receiving a low dose (1 µg/kg).^53^ Opioids can directly activate toll-like receptor-4, which results in the release and expression of inflammatory mediators including IL-1, IL-6, TNFα, and NF-κB metalloproteinase-9.^54,55^ Toll-like receptors participate in tumor-related inflammation by promoting migration of macrophages from the circulation into the tumor microenvironment and facilitating M2 polarization.^56–58^ Recently, Gjorgjevski et al. reported that the M2-like phenotype was associated with poor prognosis in patients with GBM.^59^

To the best of our knowledge, there have not been any published clinical trials or observational studies investigating the role of opioids on GBM tumor progression. While it appears that methadone does not have antitumoral capabilities at clinically nontoxic concentrations, biphalin and enkephalin should be further explored as potential GBM mediating treatments due to inconsistent findings in vitro. Further in vitro and in vivo animal testing should be performed to definitively rule out any possibility of pro-tumoral contributions before considering the introduction of these drugs into clinical settings.

### COX-2 Inhibitors

Among the many different signaling molecules involved in gliomagenesis, the cyclooxygenase-2 (COX-2) enzyme has been implicated as a main driver of malignancy.^60^ Elevated levels of COX-2 are associated with more aggressive types of tumors and worse prognoses. A study by Xu et al. demonstrated that COX-2 increases the malignancy of GBM cells via increased cell growth, invasion potential, and vascularization. This study found that the mechanism by which COX-2 increases malignancy is via downstream enhanced expression of Id1, a member of the helix-loop-helix family of transcriptional repressors.^61^ As given in Table 4, several other studies have proposed additional mechanisms and pathways by which COX-2 is involved in tumorigenesis, and have tested these effects using selective COX-2 inhibitors such as celecoxib and parecoxib. Kang et al. demonstrated that when cells were treated with celecoxib, they were more susceptible to p53-induced damage via cell-cycle arrest and autophagy.^62^

**Table 3.** Summary of Preclinical and Clinical Studies Investigating the Effects of COX-2 Inhibitor and NSAIDs on GBM Biology

Author/Year	Study Design	Pertinent Findings
Friesen (2014)	In vitro and in vivo Human GBM A172 and U118MG cells in vitro, U87MG cell line used in vivo Methadone: 10, 3, 1, µg/mL Doxorubicin: 0.1, 0.3 µg/mL	Methadone exhibits antitumoral effects by increasing the sensitivity of GBM cells to doxorubicin, at least in part by downregulation of cAMP.
Brawanski (2018)	In vitro U82 and U251 cells D,L-methadone was applied at concentrations of 0.3, 1, 15, 30, and 45 μg/mL.	Methadone exhibits antitumoral effects in GBM cells only at clinically unattainable concentrations.
Oppermann (2019)	In vitro Primary GBM and fibroblast cell cultures from 7 patients. Treated with 200 µM TMZ and/or D,L-methadone at 0, 1 nM, 10 nM, 0.1 µM, 1 µM, 5 µM, 10 µM, and 30 µM for 72 h. After that, cells were X-irradiated at a total dose of 4 Gy.	Methadone exhibits antitumoral effects in GBM cells only at clinically unattainable concentrations. Methadone does not interact with standard therapies such as TMZ and X-irradiation.
Lazarczyk (2010)	In vitro Human glioma T98G cells were treated with opioid peptide biphalin hydrochloride concentrations ranging from 50 nM to 40 µM.	Biphalin may be a better alternative to morphine due to its comparatively improved ability to inhibit human GBM cell growth.
Heiss (2009)	In vitro Neuroblastoma x glioma (NG108-15) hybrid cells were treated for 5 min with increasing concentrations of enkephalin and etorphine from 10 nM to 1 μM.	Delta-opioid receptor agonists exhibit pro-tumoral properties mediated by activation of the RTK/PI3K/Akt signaling pathway in neuroblastoma × glioma hybrid cells.

cAMP, cyclic adenosine monophosphate; GBM, glioblastoma; NG, neuroblastoma × glioma; RTK/PI3K/Akt, receptor tyrosine kinase/phosphoinositide 3-kinase/protein kinase B; TMZ, temozolomide.

**Table 4.** Summary of Preclinical and Clinical Studies Investigating the Effects of COX-2 Inhibitors and NSAIDs on GBM Biology and Patients’ Survival

Author/Year	Study Design	Pertinent Findings
Kang (2009)	In vitro Human GBM cells U87MG, U373MG, LN229, and U87MG-E6. Cells were treated with celecoxib at 8 and 30 μM for 5, 18, and 72 h.	Antitumoral properties of celecoxib are mediated at least in part by p53 modulation in human GBM cells.
Eberstal (2012)	In vitro and in vivo. N32 rat glioma cell line. Parecoxib was administered to rats at 5 mg/kg/day.	Parecoxib potentiates immunotherapy in GBM.
Suzuki (2013)	In vitro. Two human cell lines (U87MG and U251MG) and one mouse cell line (GL261) were treated with celecoxib at 10–70 μM for 48 h.	Celecoxib potentiates radiotherapy in GBM, at least in part by increasing stress on the ER.
Sharma (2011)	In vitro. Cancer stem-like cells from U87MG cells treated with 10 μM celecoxib.	Celecoxib exhibits antitumoral effects in GBM cancer stem cells independently of IL-1β.
Sareddy (2013)	In vitro. U87 and U251 cells were treated with 50–200 μM diclofenac or 20–80 μM celecoxib for 24 h.	Celecoxib and diclofenac exhibit antitumoral effects at least in part by inhibition of the Wnt/B-catenin/Tcf pathway signaling pathway.
Sareddy (2011)	In vitro. U373, GOG-C-CM, T98G, and A127 cell lines were treated with 20, 40l 60, and 80 μmol/l for 12, 24, and 36 h.	Celecoxib exhibits antitumoral effects at least in part by inhibition of the NF-κB signaling pathway.
Oksuz (2015)	In vivo (mice). C6 cell tumor bearing rats were treated with either acetaminophen 150 mg/kg, metamizole 150 mg/kg, or indomethacin 10 mg/kg for 5 consecutive days.	Acetaminophen and indomethacin exhibit antitumoral effects at least in part by inhibition of COX-3.
Bernardi (2009)	In vivo (mice) C6 glioma cells were injected into male rats and were either untreated, treated with drug-unloaded nanocapsules, treated with 1 mg/kg/day of indomethacin in solution, or treated with 1 mg/kg/day of indomethacin-loaded nanocapsules for 10 consecutive days.	Indomethacin-loaded nanocapsules exhibit greater antitumoral effects than indomethacin in solution, seen in rat GBM model.
Da Silveira (2013)	In vitro and in vivo (rats) Rat (C6) and human (U138MG, U251MG) GBM cell lines were treated with 1, 10, 50, and 100 μM ketoprofen or ketoprofen-loaded nanocapsules for 48 or 72 h. Rats implanted with C6 glioma cells received treatment with 3 mg/kg/day of ketoprofen or 3 mg/kg/day of ketoprofen-loaded nanocapsules for 15 consecutive days.	Ketoprofen-loaded nanoparticles exhibit antitumoral effects in a panel of glioma cell lines and glioma-bearing rats.
Bartels (2016)	In vitro and in vivo (mice) U87-MG, LN-18, LN-229, and U118-MG cells were treated with 0.5×, 0.75×, or 1× IC50 for 24 h. Mice were injected with U118-MG or U87-MG cells and treated with 20 mg/kg PGIA given 1×/day, 5 day/week for 12 days.	PGIA formulated in nanoparticles is a promising therapy for GBM, and can exhibit more potent antitumoral effects than traditional ibuprofen.
Wakimoto (2008)	In vitro. T98G, U118, U343, and U373 cells were 100 μmol/L of diclofenac or 10 μmol/L of meloxicam for 48 h.	NSAIDs (diclofenac and meloxicam) exhibit antitumoral effects at least in part by upregulation of 15-PGDH.
Najbauer (2015)	In vitro. U87MG and A172 cells were treated with diclofenac (0.05–0.2 mM), ibuprofen (0.5–2 mM), or ASA (0.05–0.2 mM) for 24 h.	Ibuprofen and diclofenac both exhibit antitumoral effects, but achieve this via different mechanisms.
Pantovic (2017)	In vitro. U251 MG cells were treated for 24 h with 0–1 mM indomethacin for 12, 24, and 48 h.	Indomethacin exhibits antitumoral effects at least in part via mediation of the AMPK/mTORC1 pathway.
Foulkes (2012)	In vitro. SH-SY5Y neuroblastoma cells were treated with 1 or 2 mM AAP for 24, 48, and 72 h.	Acetaminophen exhibits antitumoral properties in neuroblastoma cells at least in part by reactive oxygen species and IL-1β, along with NF-κB and p65 upregulation.
Penas-Prado (2015)	Randomized phase 2 adjuvant factorial clinical trial (*n* = 155)	Addition of thalidomide, isotretinoin, and/or celecoxib to TMZ did not improve treatment efficacy in GBM. Improved OS S was seen for triplet (20.1 months) vs doublet (17.0 months) regimens (*P* = .15). Compared with TMZ, the TMZ + isotretinoin doublet had worse PFS (10.5 vs 6.5 months, *P* = .043) and OS (21.2 vs 11.7 months, *P* = .037).
Kesari (2008)	Phase 2 clinical trial *n* = 50	Addition of thalidomide and celecoxib to TMZ did not improve PFS in GBM. Median PFS was 5.9 months and 4-month PFS was 63%. Median OS was 12.6 months, and 1-year OS was 47%.
Levin (2005)	Single-stage phase II clinical trial (*n* = 25)	Addition of celecoxib to 13-cis-retinoic acid did not improve treatment efficacy in recurrent GBM. Median PFS was 8 weeks, with a PFS at 6 months of 19%.
Grossman (2008)	Phase II clinical trial (*n* = 35) Celecoxib 400 mg was administered orally twice a day until tumor progression or dose-limiting toxicity.	In GBM patients undergoing radiation, the EIASD phenytoin has no pharmacokinetic effect on celecoxib. Estimated median survival time for all patients was 12 months.
Scheurer (2011)	Retrospective case–control study, 1534 controls and 1339 patients.	NSAIDs exhibit antitumoral effects in GBM patients, particularly in those with no history of asthma or allergies (OR = 0.80; 95% CI: 0.65, 0.99).
Egan (2016)	Case–control study. (*n* = 1123 glioma patients), (*n* = 310 (meningioma patients) and (*n* = 1296 controls)	Regular use of NSAIDs, particularly aspirin, may reduce the incidence of glioma.
Daugherty (2011)	Prospective cohort study. 302,767 individuals with 341 incident glioma cases	Regular NSAID use does not reduce risk of GBM.

AMPK/mTORC1, AMP-activated protein kinase/mammalian target of rapamycin complex; COX-3, cyclo-oxygenase-3; EIASD, enzyme-inducing antiseizure drugs; ER, endoplasmic reticulum; GBM, glioblastoma; NF-κB, nuclear factor-kappa-light-chain-enhancer of activated B cells; NSAID, nonsteroidal anti-inflammatory drug; OS, overall survival; PFS, progression-free survival; PGIA, phospho-glycerol-ibuprofen-amide; TMZ, temozolomide.

It has been shown that COX-2 inhibition via administration of parecoxib can sensitize cells to immunotherapy. These effects were seen in vitro in rat glioma cells, and in vivo in rats that showed increased survival rates after treatment with a combination of parecoxib and immunotherapy, compared with those who received immunotherapy alone.^63^ Similarly, it has been reported that treatment with celecoxib can enhance the radiosensitivity of GBM cells, at least in part by increasing stress on the endoplasmic reticulum.^64^ Additionally, celecoxib has been shown to increase apoptosis and reduce self-renewal capacity in glioma cancer stem cells.^65^ Other studies have proposed that celecoxib can suppress tumor progression via pathways that are independent of COX, including the deregulation of the Wnt/β-catenin/Tcf and NF-κB pathways.^66,67^

Due to their encouraging preclinical results indicating efficacy against GBMs, clinical studies have been done to examine how COX-2 inhibitors impact tumor progression (Table 4). Several studies have been conducted to evaluate if TMZ in combination with COX-2 inhibitors can improve patient outcomes. A phase II factorial design showed that TMZ in combination with the cytostatic drugs thalidomide, isotretinoin, and/or celecoxib did not enhance treatment efficacy compared to TMZ alone.^68^ Similarly, another phase II study looking at TMZ, thalidomide, and celecoxib combination therapy in newly diagnosed adult GBM patients found that while this triple combination was well tolerated, it did not significantly improve PFS.^69^ Levin et al. conducted a phase II clinical trial to determine if celecoxib improves outcomes in patients with recurrent GBM. Because 13-*cis*-retinoic acid (13-cRA) has previously shown promise in this particular subset of patients, the combination of this vitamin A analog with celecoxib was tested. Results showed that there was no improved efficacy of combination 13-cRA and celecoxib in the treatment of recurrent GBM, compared to 13-cRA alone.^70^

To better understand the pharmacokinetics of COX-2 inhibitors in combination with hepatic enzyme-inducing antiseizure drugs (EIASDs), Grossman et al. conducted a study to determine the effects of phenytoin on celecoxib in patients undergoing radiation therapy for GBM. Results showed that there was no clinically significant pharmacologic interaction between these 2 drugs, and that is safe to administer them in combination.^71^

### NSAIDs and Other Analgesics

In addition to COX-2 inhibitors, other nonsteroidal anti-inflammatory drugs (NSAIDs) (ie, diclofenac, ibuprofen, and meloxicam) have also been studied as a potential therapy for GBM (Table 3). These NSAIDs have been shown to exhibit their effects via COX-dependent or COX-independent mechanisms. Using an in vivo rat brain model, Oksuz et al. reported that acetaminophen and indomethacin can reduce GBM growth at least in part by inhibition of COX-3.^72^ To maximize targeted delivery of this drug class, nanoparticle systems have been developed and assessed for safety and efficacy. Indomethacin- and ketoprofen-loaded nanocapsules have both been shown to reduce tumor size in a rat glioma model.^73,74^ The common NSAID ibuprofen has been studied in the redesigned form of phospho-glycerol-ibuprofen-amide (PGIA). PGIA formulated in nanoparticles was able to provide increased efficacy in the inhibition of GBM growth in both in vivo and in vitro mice xenograft models compared to regular ibuprofen.^75^

The antitumoral properties of diclofenac and meloxicam are partly regulated via increased expression of 15-PGDH and the cell-cycle inhibitor p21.^76^ Diclofenac was also described to cause tumor inhibition by suppression of STAT-3 signaling and downstream glycolysis mediation. The same study reported that ibuprofen can reduce glioma cell proliferation and migration ability to a greater extent than diclofenac, and that these mechanisms are COX-independent as well.^77^ Indomethacin has been shown to induce cell-cycle arrest, oxidative stress, mitochondrial depolarization, and apoptosis in human glioma cells. These effects were believed to be mediated by AMPK/mTORC1, another COX-independent pathway.^78^ The common analgesic acetaminophen has been shown to decrease neuroblastoma tumor cell viability by increased production of reactive oxygen species and IL-1β, accompanied by activation of p65 and NF-κB. In vitro analysis of GBM cells with the same drug showed that this tumor cell line was more resistant to acetaminophen-induced cell damage.^79^

Similar to COX-2 inhibitors, clinical studies have looked at NSAID efficacy in GBM as well. The results of these all-encompassing NSAID studies have shown inconsistent findings. A case–control study by Scheurer et al. found that regular use of NSAIDs was associated with a preventive effect against GBM, primarily in those with no history of asthma or allergies.^80^ Similarly, another case–control study found associations that suggest that regular NSAID use, particularly aspirin, may reduce the incidence of glioma.^81^ On the other hand, a large prospective cohort study by Daugherty et al. found no evidence that regular NSAID use reduces the risk of GBM.^82^ To date, there are no studies that have investigated the perioperative use of NSAIDs or COX-2 inhibitors on glioma progression, perhaps due to the potential associated risk of perioperative bleeding. Other concerns associated with the prolonged administration of NSAIDs and COX-2 inhibitors include gastrointestinal, renal, neurological, and cardiac toxicities.^83^ In July 2015, the FDA released a warning on the chronic administration of non-aspirin NSAIDs increasing the risk for strokes and heart attacks (www.fda.gov/Drugs/DrugSafety/ucm451800.htm). Celecoxib (Celebrex) is currently the only selective COX-2 inhibitor in the United States, with an FDA-mandated black box warning for its potential gastrointestinal and cardiovascular adverse events.^84^

## Conclusion and Future Directions

GBMs are highly aggressive tumors with poor prognosis. Surgery is recommended to attain local control of the disease. Currently, there is no strong evidence to recommend changes in the anesthetic technique or analgesic care of patients undergoing GBM resection. Scalp block or wound infiltration techniques should be performed when possible to facilitate emergence from anesthesia, provide adequate perioperative pain relief, and reduce opioid use. Opioids should be given judiciously to treat severe perioperative pain. There is no convincing evidence that COX-2 inhibitors and NSAIDs should be pursued further for the treatment of GBM. Anesthesiologists should focus on the impact of anesthetics and analgesics during GBM surgery in perioperative outcomes such as pain, quality of life, rehabilitation, time to discharge, and other related metrics.

In conclusion, GBM growth and progression is a complex process. It remains poorly understood whether the cellular events triggered during surgery and anesthesia in cancer cells are diminished or amplified by adjuvant therapies, which can confound the effect of anesthetics on survival outcomes. While the in vitro effects of anesthetics on GBM pathways have been explored, these studies are difficult to replicate in vivo under circumstances that resemble craniotomies for GBM. The use of immunocompetent mice models has been proposed as a method to bridge the gap between laboratory in vitro studies and clinical studies.^85^
